# Perimenopausal and Postmenopausal Health

**DOI:** 10.1186/1472-6874-4-S1-S23

**Published:** 2004-08-25

**Authors:** Angela M Cheung, Ruhee Chaudhry, Moira Kapral, Cynthia Jackevicius, Gail Robinson

**Affiliations:** 1 University Health Network Women's Health Program, University of Toronto, 657 University Ave, Toronto, Canada; 2 University Health Network Women's Health Program, University of Toronto, 657 University Ave, Toronto, Canada; 3 University Health Network Women's Health Program, University of Toronto, 657 University Ave, Toronto, Canada; 4 University Health Network Women's Health Program, University of Toronto, 657 University Ave, Toronto, Canada; 5 Department of Psychiatry, University of Toronto, 21 King's College Circle, Toronto, Canada

## Abstract

**Health Issue:**

The average age of natural menopause in Western societies is estimated to be 51 years; women in Canada can therefore expect to live, on average, a third of their lives in post-menopausal years. During these years women are at increased risk of chronic diseases such as osteoporosis and cardiovascular disease.

**Key Findings:**

Clinical and epidemiological data on women in perimenopause are limited. There are no adequate Canadian data on symptom severity and prevalence among perimenopausal and postmenopausal women. Scientific evidence is lacking to support or refute claims that commonly used botanical products can offer therapeutic relief of menopausal symptoms.

Recent data from the Women's Health Initiative suggest that combined estrogen plus therapy increases the risk of stroke, coronary artery disease and breast cancer. Hormone therapy is no longer recommended for the prevention of chronic diseases for asymptomatic women. Stroke is an important issue for perimenopausal and postmenopausal women and sex differences may exist in the progestin treatment of stroke. Osteoporosis affects an estimated one in six women over the age of 50.

**Data Gaps and Recommendations:**

There is a need to conduct clinical and epidemiological research aimed at better understanding the menopausal transition and defining its clinical phases. Investigations aimed at alternative combinations and doses of hormone therapy and non-pharmaceutical alternatives in light of known risks and benefits are also necessary. Health care practitioners and women need to be educated on the risks and effective treatment related to cardiovascular disease so they can present for treatment more quickly and receive the most effective therapies.

## Background

The perimenopausal phase of a woman's life can span several years. The changes that occur during this period of transition will affect women to varying degrees. For some women these changes can give rise to symptoms that are severe and disruptive, and for others symptoms are mild and the transition is welcomed. Clinical and epidemiological data on women in perimenopause are limited, and what data there are come primarily from Caucasian populations. Somewhat greater attention has been given to post-menopausal women's health, and these data are often extrapolated to women in perimenopause. The average age of natural menopause in Western societies is estimated to be 51 years; women in Canada can therefore expect to live, on average, a third of their lives in post-menopausal years. During these years women are at increased risk of chronic diseases such as osteoporosis and cardiovascular disease.

## Methods

Menopause touches on a number of health issues, which are addressed separately in this chapter. For some of them new data were analyzed, and for others a review of the current knowledge is presented, in part because of the lack of menopause data in large, national surveys. Literature reviews were conducted through several databases, including MEDLINE, PREMEDLINE, PsycINFO, and EMBASE. Meta-analyses of studies published in languages other than English were included. In the case of alternative therapies, a search was done for on-line resources housed at national and international health organization web sites.

For stroke, mortality data were obtained from the Canadian Mortality Database at Health Canada on individuals aged 20 and older for whom stroke was listed as the underlying cause of death (ICD codes 431, 434, 436). Rates were age-standardized to the 1991 Canadian population. Hospital admission rates for stroke were obtained from the Canadian Morbidity Database at the Canadian Institute for Health Information. Cases were included if they were treated at an acute care hospital and had a diagnosis of stroke (ICD-9 codes 431, 434, 436). Subsequent admissions in the time period could not be identified and were not excluded. Geographic breakdowns reflect the province of hospitalization.

Information on cardiovascular disease has been supplemented by data from the FASTRAK^® ^II clinical registry, which collects quantitative and qualitative data on demographic characteristics, treatments and outcomes of all patients with acute coronary syndrome during their stay at participating Canadian hospitals. The registry includes all cardiac patients to minimize patient selection and was specifically designed to include hospitals without a tradition of participating in clinical trials in an effort to minimize hospital selection. In addition, clinical data elements are captured. Data are processed at the FASTRAK^®^ II Data Management Centre, based in the Cardiology Department of a major teaching hospital. These data were used to demonstrate practice patterns for reperfusion therapies in this setting. All female patients in the registry who had an ST elevation myocardial infarction during the period January 1, 2001, to December 31, 2001, were included. Data on 1,489 female patients were available for analysis

## Results

### Perimenopause

Natural menopause is defined as the permanent cessation of menstruation resulting from the loss of ovarian follicular activity. It is confirmed after 12 consecutive months of amenorrhea in the absence of other pathological or physiological causes, given that a woman who has experienced 12 months without flow has a 5% or lower chance of further flow.[1] Natural menopause, by this definition, can be known with certainty only in retrospect. There is currently no single biological marker to identify when a woman has reached menopause.[2] Perimenopause is defined by the World Health Organization and the North American Menopause Society as the two to eight years preceding menopause and one year following final menses.[1,3]

The average age at menopause is 51 years for Caucasian women in Western societies. Studies have reported differences in age at menopause (earlier or later) by race or country, but findings are not consistent. Studies in developing countries generally report younger age at menopause, but this is likely due to methodological differences. Factors that may affect age of menopause are not well understood, although smoking has been consistently related to earlier age at menopause by one to two years.

There is a great deal that is yet to be understood about perimenopause and women's experiences of this period of transition. Studies of menopausal symptoms are problematic, given that it is difficult to accurately identify when women enter the perimenopausal phase. Temporal associations between menopause and what may be menopausal symptoms cannot be assessed using cross-sectional studies. There have been a few good longitudinal studies that have looked at the perimenopausal transition, [4-7] including one in Manitoba. National data on symptom prevalence are not available.

A majority of women will experience menstrual changes years before their final period. Only an estimated 10% report an abrupt cessation. Heavy flow is common and, among women in the Manitoba study, led to medical consultation by about 25% of women.[6] Some women experience acute perimenopausal symptoms. The most commonly reported are vasomotor symptoms (hot flushes and night sweats), vaginal dryness, mastalgia, sleep disturbances, urinary incontinence, changes in libido, mood changes and fatigue. The prevalence of many of these symptoms varies from early to late perimenopause. Vasomotor symptoms, though they are not unique to menopause, are by far the most commonly reported. In studies of Caucasian populations, up to 85% of Caucasian women report vasomotor symptoms, and these women are more likely to report other physical and emotional symptoms. There is great variation among countries in the proportion of women reporting hot flushes. Estimates within countries also vary widely. Studies of Asian populations tend to report the lowest rates.[8] Studies in Thailand and Japan have reported rates of 6% and 12% respectively, and studies in Africa have reported rates of 30% to 80%.[8] Truly comparable studies in non-Caucasian populations are lacking.

There is some debate as to which of these symptoms can be attributed directly to hormonal fluctuations and which cannot. Short-term hormone therapy, traditionally used for the management of menopausal symptoms, has been shown to be more effective than placebo only in the treatment of vasomotor symptoms, sleep disturbances and genitourinary symptoms.[9,10] The experience of other symptoms requires further attention.

### Psychiatric Disorders in Perimenopause and Menopause

Contrary to widely held beliefs, menopause is not associated with an increase in psychiatric disorders, although the perimenopausal period may be. In one of the first studies of menopausal symptomology, Neugarten and Kraines found an increase in somatic but not in psychological complaints at menopause.[11] Multiple community studies have since indicated no increase in the prevalence of psychiatric disorders after menopause.[6,12-16] On the other hand, some studies do suggest a higher prevalence of psychiatric morbidity among perimenopausal women, especially those seeking care in menopause clinics.[17,18] Studies have found no relation between menopausal status and symptoms such as insomnia, fatigue and depression, although they have noted an increase in somatic symptoms at menopause.[11,19] There does, however, seem to be an increase in psychiatric symptoms, including depression, anxiety and psychosomatic symptoms, in the years immediately preceding the complete cessation of menses.[20] A prior history of depression has been noted to be associated with depressive and other psychological symptoms in perimenopause.[21]

Women who become depressed during the perimenopause may be reacting to one or a combination of factors. Alterations in the levels of reproductive hormones may directly affect central neurotransmitter activity and contribute to a dysregulation of the hypothalamic-pituitary-adrenal axes, leading to the onset of depression in vulnerable women.[13] Women with histories of depression, especially occurring at times of hormonal change such as premenstrually or postpartum, may be particularly vulnerable. [16,22,23] For some women, the hormone-sensitive physical complaints such as night sweats and hot flushes result in discomfort, irritability and low self-esteem that can be confused with major depressive disorders. Psychosocial stressors may also play a role. Changes in family roles, loss of fertility and fear of aging with its ensuing loss of physical attractiveness, usefulness and status in the community can lead to depression.[24] Lifestyle issues such as smoking and stress also appear to be related to symptoms of depression.[14,25]

For menopausal women experiencing mild depression as well as anxiety, insomnia and vasomotor symptoms, ovarian hormone therapy is a first-line treatment unless there are contraindications to estrogen use.[26] Amelioration of the physical symptoms may lead to an increase in well-being. In women with moderate to severe depression or anxiety, antidepressant medication is the treatment of choice and the use of estrogen in such cases remains controversial.[27,28] Studies using estrogen to treat major depression have yielded mixed results.[29,30] The use of unopposed estrogen is contraindicated in women with intact uteruses. The addition of progesterone has been associated with a worsening of mood.[29,31] The role of ovarian hormone therapy in augmenting the effects of antidepressants is still unclear.[32,33]

More research is needed to clarify the effects of using hormones in the treatment of menopausal women. Educational programs are also important to give correct information to perimenopausal women and debunk myths that may contribute to depression and low self-esteem at this time of life.[24,34] Women should be directed to some of the Web sites that provide women-centred lay information in order to further reduce their fears and misconceptions about the menopause. These include the Web sites for Menstrual Cycle Research , National Women's Health Network , Dr. Susan Love , and the Centre for Menstrual Cycle and Ovulation Research .

### Alternative Therapies

For a variety of reasons, women appear to be taking a greater interest in alternative therapies for treatment of menopausal symptoms. Despite what appears to be an increasing prevalence in use among Canadian women, very little has been published with respect to efficacy, side effects or the pharmacokinetic features of botanical products.

With the exception of black cohosh, scientific evidence is lacking about whether commonly used botanical products are effective for the treatment of menopausal symptoms. Red clover (*Trifolium pratense*) is a source of a large number of phytoestrogens (isoflavones) and has shown estrogenic activity in a number of studies; however, it has not been shown to decrease symptoms significantly more then placebo. Chasteberry (*Vitex agnus-castus*), dong quai (*Angelica sinensis*), Asian ginseng (*Panax ginseng*), North American ginseng (*Panax quinquefolius*), licorice (*Glycyrrhiza glabra*) and evening primrose (*Oenothera biennis*) are also used for the management of menopausal symptoms, but without evidence of their effectiveness. Studies have shown chasteberry to be effective in treating mastalgia and premenstrual syndrome.

A number of clinical trials, mostly German, have found that black cohosh (*Cimicifuga racemosa*) relieves perimenopausal symptoms, including hot flushes and depressed mood. Black cohosh may decrease hot flushes by 25% as compared with placebo.[35]. It has been approved for use in Germany by the Special Expert Committee of the German Federal Institute for Drugs and Medical Devices (Commission E). Phase I and phase II clinical trials are currently under way in the United States.

Many of the studies reported in the literature have been of short duration. Often an effect is observed but is similar to that seen for placebo over the short period of follow-up. It is not clear whether this relation would hold true over a longer period. Recent studies have found estrogenic activity in a number of botanical products commonly used for the treatment of menopausal symptoms, suggesting a potential for their use and a viable mechanism of action.[36]

Soy products, rich in phytoestrogens (isoflavones), have the potential to provide an exogenous source of estrogen, and the lower rates of vasomotor symptoms reported in Asian populations are sometimes attributed to greater soy intake. Data from studies considering the relation of soy, or isoflavones, to vasomotor symptoms are inconclusive. Studies in post-menopausal women, however, have shown a favourable effect on lipid levels.[2,36]

Other alternative therapies for which there is some evidence to suggest a decrease in vasomotor symptoms include relaxation, mind-body or yoga breathing strategies.[37,38] As well, topical or transdermal use of progesterone was shown in a controlled trial to decrease vasomotor symptoms in menopausal women.[39]

### Hormone Therapy

Hormone therapy (HT), or what has been known as hormone replacement therapy (HRT), is a term that describes a variety of regimens involving different estrogens and progestins. Those involving only estrogen therapy are mainly used for women without a uterus, and those involving a combination of estrogen plus progestin are for those with a uterus, since unopposed estrogen (or estrogen alone) increases the risk of endometrial cancer.[40] HT has been used for decades in the treatment of hot flushes and vaginal dryness. Over the past decade, there has been significant interest in its use for long-term prevention of chronic diseases, such as cardiovascular disease, cancer, osteoporosis and memory loss. Observational studies examining long-term hormone use have noted a slight increase in incidence of breast cancer among hormone users compared with non-users and a decrease in cardiovascular disease.[41] However, recent results from the Women's Health Initiative show an increase in both cardiovascular disease and breast cancer.[42] The reason for the differences in these findings requires further exploration.

The Women's Health Initiative (WHI) is a multi-centre, randomized trial across the United States examining a variety of factors that may affect post-menopausal health. It is the most expensive study funded by the National Institutes of Health in its history and involves 160,000 post-menopausal women.[5] One arm of this study was a randomized, double-blind placebo-controlled trial involving 16,608 post-menopausal women, which examined the use of combined estrogen plus progestin taken orally every day, as compared with placebo.[42] The study was terminated early because the risks outweighed the benefits. After an average of 5.2 years of follow-up, there was a 26% increase in breast cancer, a 29% increase in coronary heart disease and a 41% increase in stroke among hormone users. There was, however, a 34% decrease in hip fractures and a 37% decrease in colorectal cancers.

In light of these results, many scientific bodies (including the Society of Obstetricians and Gynaecologists of Canada,[43] the American College of Obstetricians and Gynecologists,[44] the North American Menopause Society,[45] the U.S. Preventive Services Task Force[46] and the Canadian Task Force on Preventive Health Care[47]) decided that HT, especially taken in this regimen, does not fit the profile of a compound for the prevention of diseases and have thus changed their recommendations. All are recommending that use of HT in post-menopausal women should be limited to the treatment of perimenopausal symptoms, the dose should be as low as possible, the duration should be as short as possible, and the use of HT should be evaluated periodically by the woman and her health care provider. These scientific bodies are also recommending against using HT for the prevention of cardiovascular diseases or as first-line therapy for the prevention and treatment of osteoporosis in asymptomatic women. The Food and Drug Administration in the United States has recently instituted a policy to include these warnings on all HT packaging, whether it is oral or transdermal, estrogen only or combined estrogen plus progestin.

Despite being the largest trial on HT to date, many questions remain unanswered. The issue of whether estrogen alone has similar effects will be addressed by an arm of the WHI that is continuing and involves 10,000 post-menopausal women. However, the trial results so far have made research on the use of HT for long-term prevention of chronic diseases much more difficult to conduct. Two further analyses were published recently from the WHI showing no difference in cognition with estrogen plus progestin therapy,[48] and if anything, HT may increase the risk of dementia in women 65 and over.[49] Furthermore, another analysis showed HT made no difference in quality of life in women without vasomotor symptoms.[50] These findings, coupled with previously reported WHI data, support the conclusion that the risks of estrogen plus progestin outweigh the benefits.

### Osteoporosis

Osteoporosis affects an estimated one in six women and one in sixteen men over the age of 50.[51] Risk factors are similar for men and women, but women experience a higher incidence of fracture, likely because of a higher prevalence of risk factors. Post-menopausal women in particular are at increased risk. In the first five years immediately after menopause, women can experience bone loss at a rate of 2% to5% per year. This rate of bone loss usually diminishes after about ten years, to 1% to 2% per year.

The most significant implications associated with osteoporosis are complications from fragility fractures. Osteoporosis is asymptomatic until a fracture occurs. Osteoporotic fractures, particularly of the hip and spine, cause considerable morbidity and mortality. For an average 50-year-old woman, the lifetime risk of a forearm fracture is 16%, of a spine fracture is 15.6%, of a hip fracture is 17.5% and of any osteoporotic fracture is more than 40%.[52] Fractures can lead to chronic pain and spinal deformity. As many as 20% of hip fracture patients will die in their first year after the fracture, often because of complications due to hospitalization[53]. Many will lose their independence and require long-term care. Men show higher rates of mortality than women following hip fracture. Whether the decrease in survival can be attributed to fractures or to comorbid illnesses is controversial.

Risk factors for osteoporotic fracture include a prior osteoporotic fracture, increasing age, low bone mineral density (BMD) and a family history of osteoporotic fracture. Other factors that may also contribute to fracture risk but may be correlated with BMD include low body weight (BMI < 20–25 kg/m2), high caffeine intake, smoking, low physical activity and low calcium intake.

A relation between BMD and fracture risk has been observed in a number of studies, and BMD remains the best quantifiable skeletal predictor of osteoporotic fracture for those who have not had a fragility fracture. The standard classification for osteoporosis and osteopenia is based on the 1993 WHO criteria,[54] which compare BMD to a reference population of post-menopausal Caucasian women. Using the WHO criteria, a BMD value greater than or equal to 2.5 standard deviations below the young adult mean is defined as osteoporosis, and a BMD value between 1 and 2.5 standard deviations below is defined as osteopenia. Women with osteopenia have a lower risk of fracture than women with osteoporosis, but they may be at greater risk than women with normal bone mineral density. The Canadian Multicentre Osteoporosis Study estimates the prevalence of osteoporosis to be 15.8% among women and 6.6% among men over the age of 50.[51] The prevalence of osteopenia among women aged 50 and over in Canada is 45.9%.[51] This is very similar to the 50% prevalence rate observed in the National Health and Nutrition Examination Survey III study in the United States.

Because osteoporotic fractures often result in loss of independence and decreased quality of life, the burden they place on women, their social supports and the health care system is substantial. An understanding of factors that contribute to fractures and strategies to prevent falls are important, especially in decreasing hip fractures among the elderly. Prevention and treatment can be started early in women at high risk of fracture. Preventive strategies currently include lifestyle modifications; ensuring adequate dietary calcium and vitamin D intake; and several pharmacological treatment options, including bisphosphonates, selective estrogen receptor modulators and, in selected cases, parathyroid hormone, calcitonin and HT. Weight-bearing exercise can increase peak bone mass in teenagers and help maintain bone mass in post-menopausal women.

### Stroke

Stroke is the fourth leading cause of death and a leading cause of disability in Canadian women.[55,56] Major risk factors for stroke include atrial fibrillation, hypertension, diabetes mellitus, smoking and hyperlipidemia. The prevalence of risk factors for stroke is similar among women and men, although women over the age of 45 are more likely to have a diagnosis of hypertension.[56] Smoking is more common among men than women in every age group except those under 20 years.[56]

**Figure 1:**
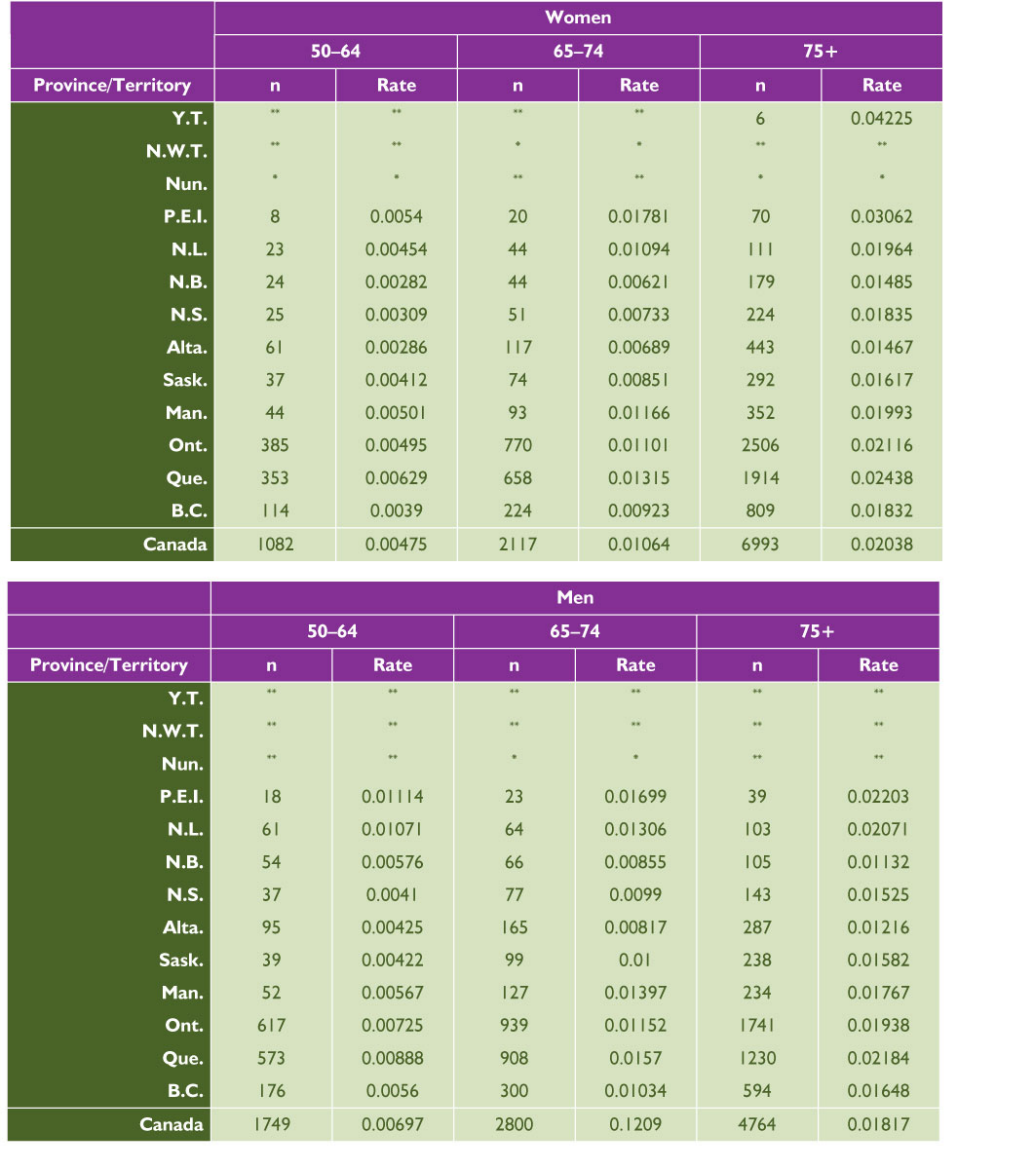
**Age and Sex-Specific Stroke Admission Rates in Canada by Province and Territory, 2000–2001. **Source: Canadian Institute for Health Information, Hospital Morbidity Database

Stroke admissions, mortality and length of stay were calculated for Canadian women and men using data from the Canadian Institute for Health Information and ICD-9 codes 431, 434 and 436. In 2000, overall hospitalization rates for stroke were lower among women than men (713 versus 955 per 100,000), but in the older age groups were higher among women (2,038 women versus 1,942 men per 100,000 aged over 75). In addition, since stroke risks increase with age and since women are overrepresented in the older age groups, the actual number of women with stroke is higher than the number of men. In Canada in 2000, for example, there were 10,592 women admitted with stroke, compared with 9,816 men. There were some variations in stroke admission rates across the country (Figure 1).

**Figure 2:**
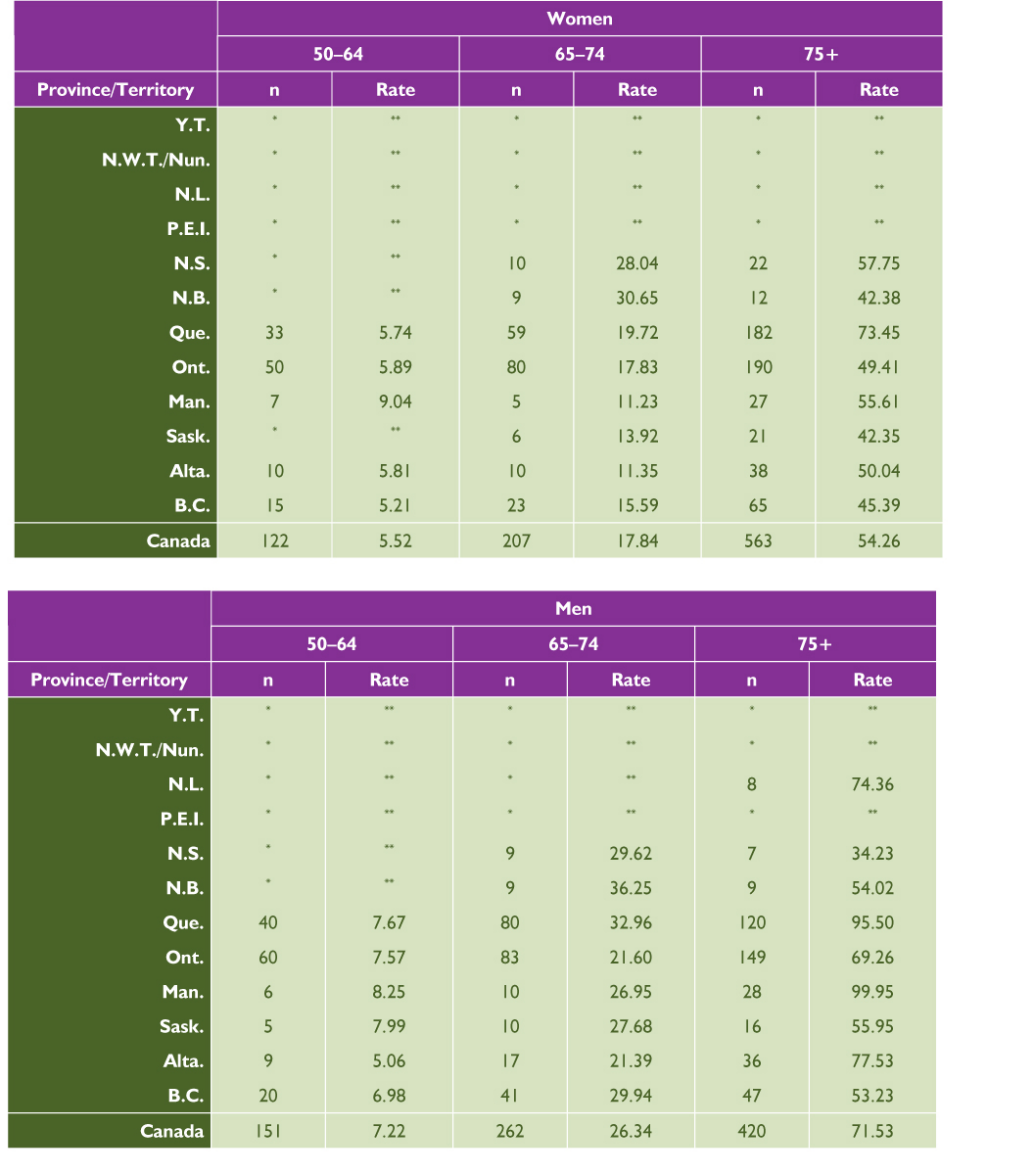
**Age and Sex-Specific Intracerebral Haemorrhage Mortality Rates per 100,000 in Canada by Province and Territory, 1997–1998 **Source: Health Canada, Canadian Mortality Database

**Figure 3:**
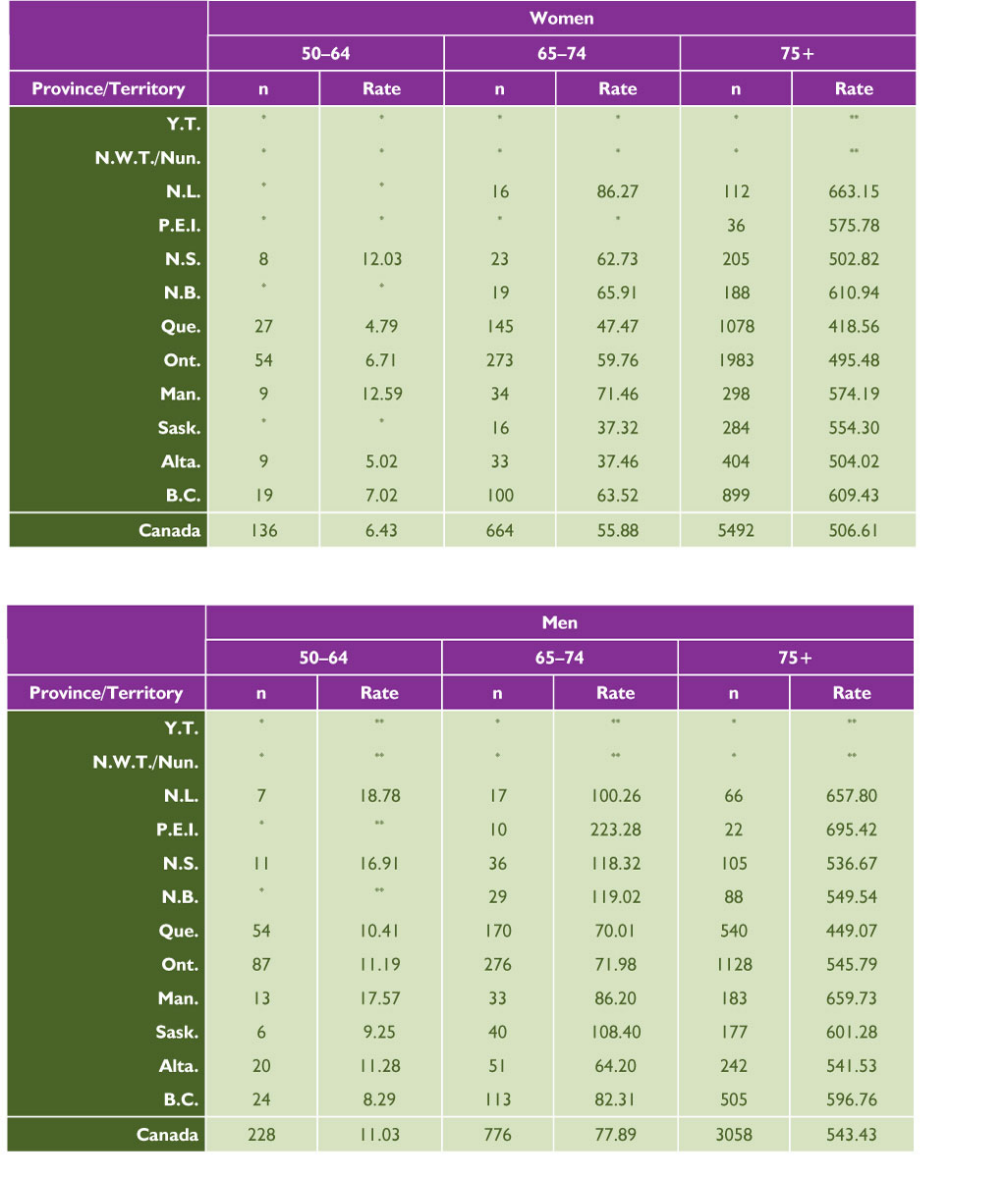
**Age and Sex-Specific Cerebral Infarction Mortality Rates per 100,000 in Canada by Province and Territory, 1997–1998 **Source: Health Canada, Canadian Mortality Database

Stroke mortality rates in Canada tend to be lower among women than men in every age group. In 1997, for example, for people aged 65 to 74 years, the mortality rate from cerebral infarction was 55.69/100,000 among women as compared with 95.87/100,000 among men. However, because of the greater proportion of women in the older age groups, in which stroke mortality is highest, stroke accounts for more deaths in women than in men (9,375 women versus 6,673 men in 1997) and a higher proportion of all deaths (9% among women versus 5.9% among men).[56] Stroke mortality rates vary across Canada (Figures 2 and 3).

Women who experience stroke are often widowed, are less likely to have social supports, and are more likely to be discharged to long-term care facilities after stroke.[57] Stroke admissions tend to be longer and more costly for women than for men ($32,000 versus $23,000 per admission).[57]

Secondary stroke prevention includes antiplatelet agents, anticoagulants for atrial fibrillation, and carotid endarterectomy for carotid stenosis. Canadian data suggest that sex differences exist in the secondary prevention of stroke. For example, older women (aged over 85) are less likely than men to be prescribed antiplatelet agents.[58] In addition, Canadian women are about half as likely as men to undergo carotid endarterectomy, even after adjustment for age.[57,59]

### Cardiovascular Disease

Cardiovascular disease (CVD) is the leading cause of death in Canadian women.[55,56] In general, major risk factors for CVD include hypertension, dyslipidemia, diabetes mellitus, smoking, inactivity, male sex and older age. According to the National Population Health Survey 1996–1997 analyses published in *The Changing Face of Heart Disease and Stroke in Canada *(2000),[56] many of the major risk factors have similar prevalence among men and women. However, more women are likely to have hypertension, and more men than women are likely to have diabetes mellitus and to be smokers.

While CVD has been well accepted as a prominent condition in men, there has been some controversy about the signs and symptoms, diagnosis and treatment of CVD in women. It is under-appreciated that women may present with a broader range of signs and symptoms of heart disease than men.[60,61] Sex differences have been found in the management of CVD. Physicians pursue a less aggressive management approach to CVD for women, who receive fewer referrals for diagnostic and revascularization procedures and are under-treated with effective medications. [62-66]

Preferential prescribing for males has also been uncovered for evidence-based therapies, Canada and the United States showing similar patterns. In a cohort of 2,070 post-myocardial infarction (MI) patients from Alberta and Nova Scotia followed from 1987 to 1992, women not only had a significantly lower rate of prescription for Aspirin (69% versus 79%), beta-blockers (36% versus 48%) and thrombolytic agents (20% versus 30%), but also a higher mortality rate than their male counterparts (18% versus 12%).[67] Several other studies in North America throughout the 1990s support these findings of differential prescribing according to sex in the treatment of cardiovascular disease. [68-71]

This is of particular concern, since the beneficial effects of therapy for acute coronary syndromes – such as fibrinolytics, acetylsalicylic acid (ASA), beta-blockers and angiotensin-converting enzyme inhibitors – appear to be equally effective in women as in men. [72-74] Many of these studies includeonly about 25% of women as participants, but the benefits of therapy are of a similar magnitudeas in men.

More recently, Haddad and colleagues studied a small group of 717 MI patients in Nova Scotia to assess their medical management and found that there were few differences in prescribing patterns between men and women.[75] Previous sex differences may be decreasing, but ineffective or potentially harmful therapies are still being preferentially given to women. Most recently, the Institute for Clinical Evaluative Sciences (ICES) in Ontario assessed the use of medication in older post-MI patients (65+ years) over the period of 1994–1995 to 1996–1997 and found few sex-related differences in treatment rates.[76] Overall, there was room for improvement in the use of evidence-based therapies in both sexes. However, there was a 4% to 5% higher rate of use of calcium channel blockers among women aged 65 to 74 and 75 to 84 years as compared with men. This class of drug has not been shown to improve mortality after MI and in some case may increase mortality. In other studies, women have received more nitrates and calcium channel blockers, despite the fact that these drugs have not been shown to decrease mortality.[77,78] In a study assessing whether evidence changes practice, using the uptake of the landmark 4S statin trial into practice as the example, the increase in the rate of statin therapy among women was 1.6-fold lower than that among men (*p *= 0.006). This study indicates a lag in the use of evidence-based therapies – in this case, statins – in older women in Ontario.[79]

**Figure 4:**
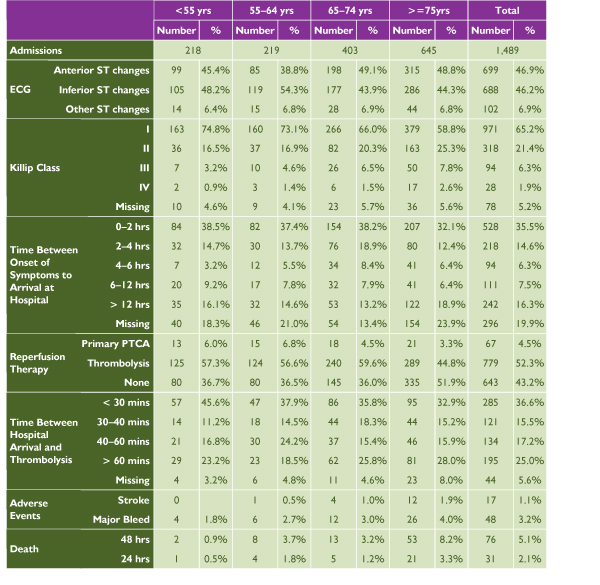
**Use of Reperfusion Therapy in Women with ST Elevation Acute Myocardial Infarction in Canada, January 1, 2001 to December 31, 2001 **Source: FASTRAK^®^11 Clinical Registry, 2001

**Figure 5:**
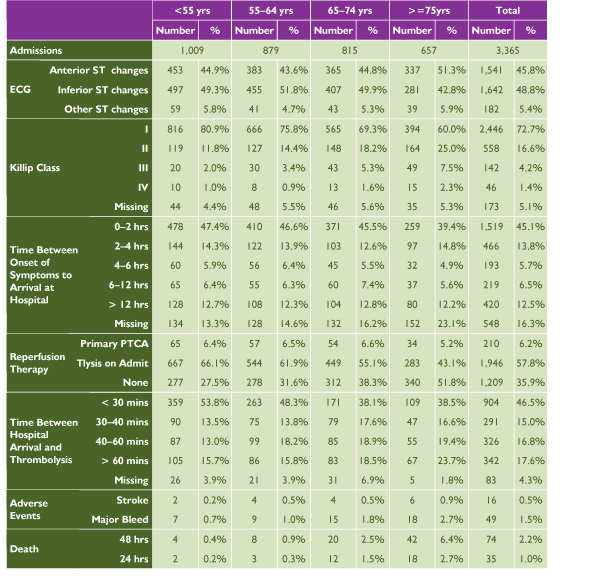
**Use of Reperfusion Therapy in Men with ST Elevation Acute Myocardial Infarction in Canada, January 1, 2001 to December 31, 2001. **Source: FASTRAK^®^11 Clinical Registry, 2001

The FASTRAK II database of acute cardiac hospital admissions across Canada for the year 2001 was used to determine more recent trends by sex in the presentation of acute myocardial infarction (AMI) and use of acute therapies. There were 4,897 patients with AMI in 2001, 3,365 (69.3%) of whom were male and 1,489 (30.7%) of whom were female. Figure 4 summarizes the AMI data for females, and Figure 5 summarizes the data for men. Women take longer than men to present to hospital after the start of their symptoms and are more ill upon presentation (Killip class). Fewer women in the younger age groups (< 55 years and 55 to 64 years) appear to receive reperfusion therapy with either fibrinolysis or primary angioplasty. Fewer women in the older age groups (65 to 74 years and over 75 years) receive primary angioplasty. In all age categories, women have a longer time from arrival to receipt of fibrinolysis. The target goal of door-to-needle time of under 30 minutes is reached in 48.5% of men versus only 38.8% of women. While the numbers are small, more women than men have strokes and major bleeding, and the rate of death in hospital at 48 hours after admission is higher among women across allage categories. These data show that women present later and are more ill at presentation, are treated less often and later with fibrinolysis and angioplasty, and have more complications of their AMI and its treatment.

Sex differences in prescribing patterns for congestive heart failure have not been consistent, but they tend to be less than those found in the multitude of studies evaluating AMI.[80,81] In an ICES analysis of older patients with congestive heart failure in Ontario from 1994–1995 to 1996–1997, no sex-related differences were found in the rates of use of ACE inhibitors.[76] One Canadian study, which assessed the use of thromboembolic prophylaxis in 3,575 patients with atrial fibrillation from 12 hospitals during the period 1993–1994, found that significantly more females received ASA or no treatment as compared with men. Warfarin was the drug of choice for atrial fibrillation in this study.[82] It is possible that some elderly, frail women may have contraindications that prevent them from receiving the more effective therapy, warfarin.

Greater awareness and reduction of risk factors for cardiovascular disease in women are needed. While preventive medications are being used more frequently in women, greater use of effective therapies and less use of ineffective or potentially harmful ones are warranted.

## Summary of Results

• Clinical and epidemiologic data on women in perimenopause are limited. There do not appear to be adequate Canadian data on symptom severity and prevalence among perimenopausal and post-menopausal women. Existing data on age at menopause and experience of the menopausal transition come primarily from Caucasian populations.

• Perimenopausal and post-menopausal women differ hormonally and experientially. Therapies tested on one population should not necessarily be extrapolated to the other.

• Scientific evidence is lacking to support or refute claims that commonly used botanical products can offer therapeutic relief of menopausal symptoms. There are inadequate data on the efficacy, side effects and pharmacokinetic features of these products. However, recent studies of botanical products commonly used for treatment of menopausal symptoms have demonstrated estrogenic activity, suggesting a potential for their use and a viable mechanism of action.

• Recent data from the Women's Health Initiative suggest that combination HT increases the risk of stroke, coronary artery disease and breast cancer. The study did demonstrate a decrease in risk of colon cancer and hip fracture, but no difference in cognition or quality of life in asymptomatic women. Thus, combination HT is no longer recommended for prevention of chronic diseases in asymptomatic women.

• Sex differences may exist in the secondary prevention of stroke, and there may be evidence to suggest differential prescribing for CVD according to sex.

• Stroke is an important issue for women, particularly those in the older age groups. Women may be less likely than men to receive antiplatelet agents and carotid endarterectomy for secondary stroke prevention, although further research is needed to determine whether this is due to sex alone, to age or to other clinical factors.

• Women are at greater risk of osteoporosis and osteoporotic fractures than men. However, women have lower in-hospital mortality rates than men following hip fracture.

• Psychiatric disorders are not significantly increased among menopausal women, although there is some increase in psychiatric symptoms in the perimenopause.

## Discussion

### Recommendations

• Conduct clinical and epidemiologic research aimed at better understanding the menopausal transition and defining its clinical phases. In order to collect data on perimenopausal women, we must be able to identify them. Age is often used as a surrogate for menopausal status, making it difficult to differentiate conditions that may be due to biological changes from those that may be attributed to other factors.

• Investigate alternative combinations and dosing of hormone therapy, in light of known risks and benefits, with respect to treatment of menopausal symptoms and long-term outcomes.

• Investigate non-pharmacological alternatives (risks and benefits) for the treatment of menopausal symptoms, particularly those alternatives already in common use.

• Educate health care practitioners and women about the risks and effective treatments related toCVD so that women may present for treatment more quickly and receive the most effective therapies.

• Investigate the use of ovarian hormones to augment the effect of antidepressants in menopausal women.

## Note

Views expressed in this report do not necessarily represent the views of the Canadian Population Health Initiative, the Canadian Institute for Health Information or Health Canada.
